# The tumour-suppressive *miR-29a/b1* cluster is regulated by CEBPA and blocked in human AML

**DOI:** 10.1038/sj.bjc.6605751

**Published:** 2010-07-13

**Authors:** M Eyholzer, S Schmid, L Wilkens, B U Mueller, T Pabst

**Affiliations:** 1 Department of Clinical Research, University of Bern, Bern, Switzerland; 2 Department of Medical Oncology, Inselspital, Bern University Hospital, University of Bern, Bern, Switzerland; 3 Department of Pathology, Hospitals of the Region Hannover, Hannover, Germany; 4 Department of Internal Medicine, Bern University Hospital, University of Bern, Bern, Switzerland

**Keywords:** AML, CEBPA, *miR-29a/b/c* family, transcriptional regulation

## Abstract

**Background:**

CCAAT/enhancer-binding protein-*α* (CEBPA) is crucial for normal granulopoiesis and is frequently disrupted in acute myeloid leukaemia (AML). Increasing evidence suggests that CEBPA exerts its effects, in parts, by regulating specific microRNAs (miRNAs), as previously shown for *miR-223*. The aim of this study was to investigate the genome-wide pattern of miRNAs regulated by CEBPA in myeloid cells.

**Methods:**

In Kasumi-1 cells, conditionally expressing CEBPA, we assessed the expression of 470 human miRNAs by microarray analysis. We further investigated the microarray results by qRT-PCR, luciferase reporter assays, and chromatin immunoprecipitation assays.

**Results:**

In all, 18 miRNAs were more than two-fold suppressed or induced after CEBPA restoration. Among these 18 miRNAs, we focused on CEBPA-mediated regulation of the tumour-suppressive *miR-29b*. We observed that *miR-29b* is suppressed in AML patients with impaired CEBPA function or loss of chromosome 7q. We found that CEBPA selectively regulates *miR-29b* expression on its *miR-29a/b1* locus on chromosome 7q32.3, whereas *miR-29b2/c* on chromosome 1q32.2 is not affected.

**Conclusion:**

This study reports the activation of the tumour-suppressive *miR-29b* by the haematopoietic key transcription factor CEBPA. Our data provide a rationale for *miR-29b* suppression in AML patients with loss of chromosome 7q or CEBPA deficiency.

Haematopoiesis is a highly orchestrated interaction of lineage-specific transcription factors driving pluripotent precursor cells to differentiate towards mature blood cells (Rosenbauer and Tenen, 2007). Increasing evidence suggests that this differentiation along the various haematopoietic lineages is, in part, also regulated by microRNAs (miRNAs) (Lawrie, 2007; Garzon and Croce, 2008; Pelosi *et al*, 2009). miRNAs are small, non-coding RNAs, which silence target genes by base-pairing to untranslated mRNA regions. Thereby, miRNAs adjust expression of specific transcription factors in a post-transcriptional manner (Shivdasani, 2006; Ambros and Chen, 2007). Deregulation of either haematopoietic transcription factors or miRNAs is a common event in the molecular pathogenesis of human leukaemias (Tenen, 2003; Kluiver *et al*, 2006; Rosenbauer and Tenen, 2007; Fabbri *et al*, 2008).

One of the key transcription factors for normal haematopoiesis is the CCAAT/enhancer-binding protein-*α* (CEBPA). It has been shown to be crucial for myeloid differentiation towards mature granulocytes (Zhang *et al*, 1997; Radomska *et al*, 1998). In human acute myeloid leukaemia (AML), CEBPA function is frequently disrupted (Pabst and Mueller, 2007). Approximately 10% of AML patients show dominant-negative mutations in the *CEBPA* coding region (Pabst *et al*, 2001b). In addition, CEBPA expression is suppressed by the leukaemogenic fusion proteins AML1-ETO, AML1-MDS1-EVI1, or CBFB-SMMHC in AML patients bearing the chromosomal rearrangements t(8;21), t(3;21) or inv(16) respectively (Pabst *et al*, 2001a; Helbling *et al*, 2004, 2005).

During normal haematopoiesis, various CEBPA downstream effectors have been described (Tenen, 2003; Mueller and Pabst, 2006), including so far at least one miRNA (*miR-223*) (Fazi *et al*, 2005; Fukao *et al*, 2007; Eyholzer *et al*, 2009). The activation of *miR-223* by CEBPA can trigger neutrophil differentiation and is necessary for maintaining proper function of mature neutrophils (Fazi *et al*, 2005, 2007; Johnnidis *et al*, 2008). On the basis of these reports and the prominent role of CEBPA for normal myelopoiesis, we assessed in this study the genome-wide regulation of miRNAs by CEBPA in myeloid leukaemic cells. We screened 470 human miRNAs for their expression levels in CEBPA-deficient leukaemic Kasumi-1 cells using a conditional CEBPA expression system. We identified 18 miRNAs whose expression levels changed more than two-fold after CEBPA induction. Among them, we identified the tumour-suppressive *miR-29a/b1* cluster to be a direct target of CEBPA.

## Patients and methods

### Patients, controls and cell lines

Bone marrow samples from 66 consecutive AML patients collected at diagnosis before treatment were used, comprising all FAB subtypes. Leukaemic cells were collected using Ficoll gradient (Lymphoprep; Axis-Shield PoC AS, Oslo, Norway). miRNA was extracted using the miRNeasy Mini kit no. 217004 (Qiagen AG, Hombrechtikon, Switzerland). Mature monocytes or granulocytes from six healthy volunteers were isolated from peripheral blood using the EasySep selection kits nos. 18088-CD14 and 18682-CD66b (RoboSep; StemCell Technologies, Vancouver, Canada). CD34+ myeloid stem cells from three patients were enriched using the CliniMacs CD34 Complete kit no. 177–01 (Miltenyi Biotec, Auburn, CA, USA). Informed consent from patients and volunteers was obtained according to the Declaration of Helsinki Principles. Clinical characteristics are summarised in Supplementary Table S1 (Supplementary Material).

Leukaemic Kasumi-1 cells stably transfected with an inducible *CEBPA*-*oestrogen receptor* (*ER*) fusion construct (*CEBPA-ER*) (Pabst *et al*, 2001a) were cultured in phenol red-free RPMI 1640 supplemented with 10% foetal calf serum (FCS). The CEBPA-ER fusion protein was activated using 1 *μ*M *β*-oestradiol. All reagents were from Sigma-Aldrich (Buchs, Switzerland).

Leukaemic U937 cells stably transfected with the tetracycline-inducible (tet-off) oncogenic t(8;21) fusion protein AML1-ETO (Pabst *et al*, 2001a) were cultured in RPMI 1640 supplemented with 10% FCS and 0.75 *μ*g ml^−1^ tetracycline. To induce AML1-ETO expression the cells were extensively washed with PBS and cultured in RPMI 1640, supplemented with 10% tetracycline-free FCS (PAA Laboratories GmbH, Pasching, Austria).

Leukaemic HL60, K562, Kasumi-1 and U937 cells, and H1299 lung cancer cells (ATCC, Manassas VA, USA) were cultured in RPMI 1640 with 10% FCS. The cell lines were characterised by molecular diagnostics and cytogenetics, and cell morphology was monitored by microscopy according to ATCC guidelines (http://www.atcc.org >cultures and products > technical support > technical literature > technical bulletin no. 8). No abnormalities in cell morphology were observed in these cell lines, both at low and high densities of cultures during the course of these experiments. Repetitive mycoplasma screening remained negative in these cell lines (PCR mycoplasma test kit Promokine no. PK-CA91–1048; PromoCell GmbH, Heidelberg, Germany).

### miRNA microarray

Parental Kasumi-1 and Kasumi-1 cells with the inducible *CEBPA-ER* construct were collected before and 72 h after *β*-oestradiol treatment. miRNA was extracted using the miRNeasy mini kit no. 217004 (Qiagen AG), and miRNA quality was determined using Agilent 2100 Bioanalyzer (Agilent Technologies, Basel, Switzerland).

To assess miRNA expression profiles, we used the human miRNA microarray kit no. G4470A, detecting 470 human and 64 viral miRNAs based on the Sanger database version 9.1 (http://www.mirbase.org). Scanning and image analysis were carried out using the Agilent DNA microarray scanner (no. G2565BA; XDR 5/100, single pass, green). Feature Extraction software (version 9.5; Agilent Technologies) was used for data extraction from raw microarray image files using the miRNA_v1_95_May07 FE protocol (grid 016436_D_20070426). Data analysis was carried out using GeneSpring GX 9.0 (Agilent Technologies) expression analysis software and expression values were corrected for oestrogen effects. The cut-off for miRNA candidates was set at more than two-fold changes in expression (suppression or induction) after CEBPA restoration, and such changes had to be observed in two independant experiments. The microarray kit, equipment and software used for these arrays were from Agilent Technologies.

### Luciferase reporter assay

The human DNA sequence comprising −682 to +296 bp upstream of the primary *miR-29a/b1* transcription start site (GenBank accession number EU154353) was cloned into the pGL3b luciferase vector using *Kpn*I and *Nhe*I restriction sites. This construct was co-transfected with a human *CEBPA* expression plasmid (pcDNA3) in H1299 cells using Lipofectamine 2000 (Invitrogen, Basel, Switzerland). Luminescence was detected using the Dual-Luciferase Reporter Assay (Promega, Dübendorf, Switzerland). Primer sequences are indicated in Supplementary Table S2 (Supplementary Material).

### Quantitative RT-PCR

*miR-29b* expression in samples from AML patients and healthy volunteers was assessed using the miScript SYBR Green PCR kit no. 218073 and primer assay hs-miR-29b no. MS_6566 (Qiagen AG). Expression values were normalised to the geometric mean (Peltier and Latham, 2008) of *miR-93* and *miR-191* expression (nos. MS_3346 and MS_3682 respectively; Qiagen AG). To distinguish between *miR-29a* and *miR-29c* expression, we used TaqMan microRNA assays no. 001212 (29a) and no. 000578 (29c) and TaqMan universal PCR master mix No AmpErase UNG no. 4324018 (Applied Biosystems, Rotkreuz, Switzerland). Primer sequences for *pri-miR-29a/b/c* detection using QuantiTect SYBR Green PCR kit no. 204143 (Qiagen AG) are indicated in Supplementary Table S2 (Supplementary Material). Expression values of *miR-29a/b/c* and their primary transcripts in cell line experiments were normalised to *miR-93* expression, as *miR-93* showed robust and stable expression during the time courses in this study. All qRT-PCR reactions were carried out on 7900HT Fast Real-Time PCR system (Applied Biosystems).

### Chromatin immunoprecipitation assay

Chromatin immunoprecipitation assays were performed using the ChIP-IT Express Enzymatic kit no. 53009 (Active Motif, Rixensart, Belgium). Immunoprecipitation of sheared chromatin of parental U937 as well as of Kasumi-1-CEBPA-ER cells collected 72 h after *β*-oestradiol treatment was performed using antibodies against CEBPA (polyclonal rabbit IgG, sc-61X), polymerase II (sc-900X), and rabbit IgG (sc-2027; all from Santa Cruz, Heidelberg, Germany). Sequences of the PCR primers to detect CEBPA binding to the *pri-miR-29a/b1* promoter or to the *pre-miR-223* regulatory element as positive control (Fazi *et al*, 2005) are described in Supplementary Table S2 (Supplementary Material).

### Western blot analysis

Protein detection was carried out from whole-cell lysates using antibodies against CEBPA no. 39306 (1:500; Active Motif) and DNMT3B sc-10236 (1:500; Santa Cruz). For loading control, *β*-actin antibody MAB1501 (1:10^5^; Chemicon/Milipore, Zug, Switzerland) was used. Horseradish-peroxidase-linked secondary antibodies (1:5000 each) were: anti-mouse no. NA931V, anti-rabbit no. NA934V (Amersham, GE Healthcare Bio-sciences, Uppsala, Sweden), and anti-goat sc-2020 (Santa Cruz).

### URL and statistical analysis

Conservation studies of the *pri-miR-29a/b1* (GenBank accession number EU154353) and *pri-miR29b2/c* (EU154351 and EU154352) loci were carried out using http://www.genome.ucsc.edu/ (assembly March 2006). Promoter analysis for putative CEBP binding sites were performed using Genomatix MatInspector software, release 7.7(3) (Genomatix Software GmbH, Munich, Germany). Differences in promoter activities and *miR-29* expression levels were analysed by *t*-test, with *P*<0.05 defining significance using GraphPad Prism software version 4.0 (GraphPad Software Inc., La Jolla, CA, USA).

## Results

### Genome-wide changes in miRNA expression after CEBPA restoration in human AML

To identify miRNAs regulated by the myeloid key transcription factor CEBPA in the haematopoietic system, we carried out miRNA microarrays assessing 470 human miRNAs. We used leukaemic Kasumi-1 cells lacking detectable amounts of endogenous CEBPA, however, containing an inducible *CEBPA*-ER fusion construct (Pabst *et al*, 2001a). We treated these cells with *β*-oestradiol for 72 h to restore the CEBPA function, and analysed the changes in miRNA expression. We found that the expression of 18 miRNAs changed more than two-fold after restoring CEBPA function compared with untreated Kasumi-1-CEBPA-ER cells and after exclusion of effects because of oestrogen treatment (Table 1). Of the 18 miRNAs, 8 (44%) were suppressed (Table 1A: *miR-98*, *miR-181b*, *miR-197*, *miR-210*, *miR-342*, *miR-432*, *miR-550*, and *miR-776*), whereas 10 miRNAs (56%) were induced (Table 1B: *miR-29b*, *miR-223*, *miR-370*, *miR-496*, *miR-572*, *miR-575 miR-630*, *miR-638*, *miR-663*, and *miR-765*; Supplementary Table S3). *miR-223*, a previously identified target of CEBPA (Fazi *et al*, 2005; Fukao *et al*, 2007; Eyholzer *et al*, 2009), was confirmed and used as a positive control for the array experiments, with a two-fold induction after restoring CEBPA in our cell line model.

With a focus on haematopoiesis, differentiation, and/or carcinogenesis, we summarised the rapidly increasing literature available for the 18 identified miRNAs in Table 1 (for references see also Supplementary Table S3). Most of these reports describe expression patterns in various types of normal tissues and cancer, whereas reports on the regulation of specific miRNAs are rare.

In this study, we focused on CEBPA-regulated miRNAs with tumour-suppressive functions in haematopoiesis, and *miR-29b* represented the most prominent candidate. *miR-29b* belongs to a miRNA family comprising three members (*miR-29a*, *-29b*, and *-29c*), which have been reported to be suppressed in various cancer types (Fabbri *et al*, 2007; Mott *et al*, 2007; Wang *et al*, 2008), including leukaemias (Li *et al*, 2008; Stamatopoulos *et al*, 2009; Garzon *et al*, 2009b). Furthermore, they were shown to induce differentiation (Wang *et al*, 2008; Li *et al*, 2009; Garzon *et al*, 2009b) and apoptosis (Park *et al*, 2009), and inhibit epigenetic silencing due to *de novo* methylation (Fabbri *et al*, 2007; Garzon *et al*, 2009b).

### CEBPA mediates *miR-29b* expression in AML

**Figure 1:**
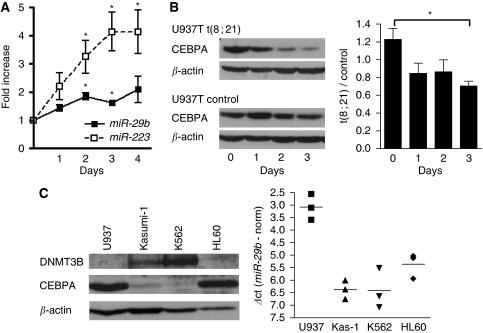
CEBPA mediates *miR-29b* expression in leukaemic cells. (**A**) Mature *miR-29b* expression was induced two-fold after restoring CEBPA function in leukemic Kasumi-1 cells. The CEBPA target *miR-223* was used as positive control for restored CEBPA function. Results are given as fold expression compared with untreated cells (day 0, ^*^P<0.05) and represent three independant experiments. (**B**) Conditional expression of AML1-ETO abolished CEBPA protein expression in U937 leukemic cells (**B**, left) and suppressed *miR-29b* expression (^*^P<0.05; **B**, right). *miR-29b* expression is given as fold changes compared with the control cells U937-T (ΔΔ*C*_t_-method) and represent three independant experiments. (**C**) Protein levels of the putative *miR-29b* activator CEBPA and the *miR-29b* target DNMT3B inversely correlated in leukemic cell lines (**C**, left). *miR-29b* expression correlated to CEBPA, and inversely correlated to DNMT3B protein levels (**C**, right). *miR-29b* expression is given as Δ*C*_t_-values (*C*_t_(*miR-29b*)−*C*_t_(normalisation)) representing three independant experiments.

We aimed to verify the results of the miRNA microarray by qRT-PCR. We observed that *miR-29b* was, indeed, induced two-fold after CEBPA restoration in the Kasumi-1-CEBPA-ER cell line system (Figure 1A). We then investigated the effect of CEBPA knock down on miR-29b expression. We used parental U937 leukaemic cells, expressing high levels of endogenous CEBPA as well as a tet-off system conditionally expressing the oncogenic t(8;21) fusion protein AML1-ETO (Pabst *et al*, 2001a). Induction of AML1-ETO in these cells efficiently blocked CEBPA protein expression (Figure 1B, left), which led to suppressed *miR-29b* expression (Figure 1B, right).

Interestingly, CEBPA-associated expression of *miR-29b* was further observed across a variety of leukaemic cell lines: the expression of CEBPA protein and *miR-29b* in HL60, K562, Kasumi-1, and U937 cells inversely correlated to the protein expression of the previously identified *miR-29b* target gene DNA methyltransferase 3B (DNMT3B, Figure 1C; Fabbri *et al*, 2007; Garzon *et al*, 2009b). As DNMT3B is mediating *de novo* DNA methylation and thus epigenetically inactivates tumour suppressor genes in cancer (Robertson *et al*, 1999; Rhee *et al*, 2002; Lin *et al*, 2007), these observations connect blocked differentiation through CEBPA suppression with deregulated methylation because of the suppressed *miR-29b* activity.

### *miR-29b* expression is suppressed in AML patients with impaired CEBPA function or with monosomy 7 or del(7q)

**Figure 2:**
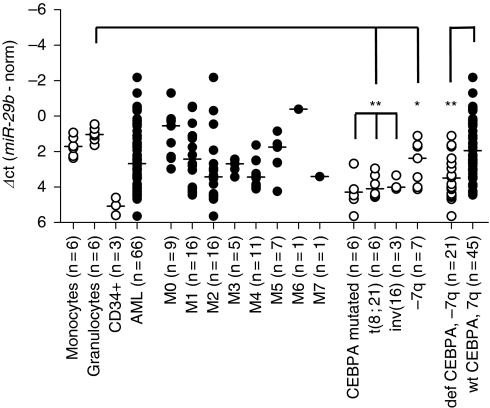
*miR-29b* expression in AML patients and healthy controls. *miR-29b* expression was assessed in samples from 66 AML patients, in three samples of enriched CD34+ myeloid stem cells as well as in mature granulocytes and monocytes from six healthy volunteers. The cohort of consecutive AML samples showed suppressed *miR-29b* expression compared with granulocytes (*P*=0.043). Patients with CEBPA deficiency (*CEBPA* mutations, t(8;21), inv(16)) or chromosome 7q alterations (monosomy 7 or del(7q)) represented roughly one-third of the entire cohort and showed differences in *miR-29b* expression compared with granulocytes from healthy volunteers (^**^P<0.001 for all three subgroups with deficient CEBPA function, and ^*^P<0.05 for −7q) as well as compared with the remaining 45 AML patients (wt CEBPA and 7q, ^**^P<0.001). *miR-29b* expression was not suppressed in the remaining 45 patients (wt CEBPA and 7q) if compared with mature granulocytes (*P*=0.182, NS). Expression levels are given as Δ*C*_t_-values (*C*_t_(*miR-29b*)−*C*_t_(normalisation)).

To evaluate the importance of CEBPA-mediated *miR-29b* induction *in vivo*, we analysed *miR-29b* expression in diagnostic samples of 66 AML patients, three samples of enriched CD34+ myeloid stem cells, and in samples of mature granulocytes and monocytes from 6 healthy volunteers (Figure 2).

We observed that the mean expression of *miR-29b* was suppressed in the entire cohort of AML patients compared with normal granulocytes (*P*=0.043). In our cohort, we then separately analysed the patients with suppressed CEBPA function. This group comprised the AML patients with *CEBPA* mutations, with t(8;21) or with inv(16) (Pabst *et al*, 2001a, 2001b; Helbling *et al*, 2005). We found that these AML patients had suppressed *miR-29b* compared with mature granulocytes (*P*=0.0001 for *CEBPA* mutated, *P*<0.0001 for t(8;21) and inv(16)). Remarkably, the low *miR-29b* levels were comparable with *miR-29b* expression in CD34+ precursor cells, which hardly express detectable amounts of CEBPA (Radomska *et al*, 1998).

In addition, we confirmed previous observations (Garzon *et al*, 2009a) that *miR-29b* is suppressed in patients with monosomy 7 or del(7q) (*P*=0.012). On combining AML patients with alterations of chromosome 7q or CEBPA (*n*=21), we observed low *miR-29b* expression compared with the remaining 45 patients of our cohort, with other or no detectable genomic alterations (*P*=0.0002). We thus confirmed in our cohort of 66 AML patients that *miR-29b* expression is associated with CEBPA levels and therefore suppressed in patients with disrupted CEBPA function.

### Only *miR-29a/b1* is induced after restoring CEBPA in human AML cells

**Figure 3:**
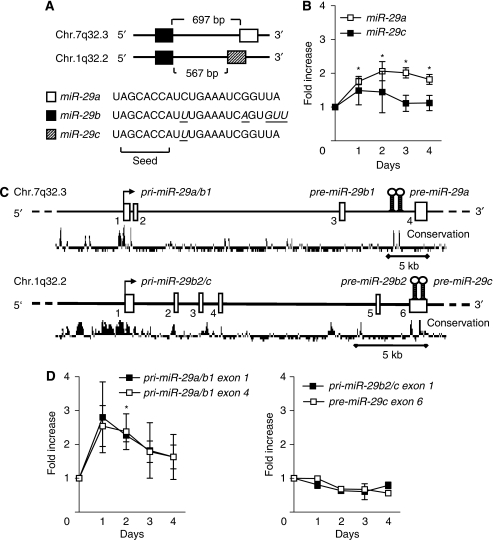
CEBPA activates *miR-29b* at the *pri-miR-29a/b1* locus on chromosome 7q32.3. (**A**) *miR-29b* belongs to a family of three members (a, b, c), encoded on two loci on chromosome 1q32.2 and chromosome 7q32.3. Although *miR-29b* is encoded on both chromosomes, the mature *miR-29b* sequence encoded from the two loci is identical. (**B**) In addition to *miR-29b*, only mature *miR-29a* is induced after CEBPA restoration, but not mature *miR-29c*. (**C**) *miR-29* family members are generated from two polymerase II primary transcripts: *pri-miR-29a/b1* (GenBank accession number EU154353) and *pri-miR-29b2/c* (EU154351 and EU154352). These primary transcripts are processed to double stranded precursors (*pre-miR-29*) and ultimately to the mature single stranded *miR-29*. Both *pri-miR-29s* are highly conserved in their putative promoter region and in the *pre-miR-29* stem sequences, encoded in the last intron (*pre-miR-29a, -b1*) on chr.7q32.3 and the last exon (*pre-miR-29b2, -c*) on chr.1q32.2 respectively. (**D**) Primer pairs dispersed over the *pri-miR-29a/b1* and *pri-miR-29b2/c* confirmed induction of the *pri-miR-29a/b1* locus on chr.7q32.3 (**D**, left), whereas the *pri-miR-29b2/c* on chromosome 1.q32.2 is not affected by CEBPA expression (**D**, right). Results are given as fold expression compared with untreated cells (day 0, ^*^P<0.05) and represent three independant experiments.

*miR-29b* belongs to the *miR-29* family that is encoded in two clusters on two chromosomes (Figure 3A): *miR-29a* as well as *miR-29b* on chromosome 7q32.3, and *miR-29c* as well as, again, *miR-29b* on chromosome 1q32.2. Mature *miR-29b* is therefore encoded by two distinct precursor stem sequences (pre-miRNA) on both chromosomes, a *pre-miR-29b1* and *pre-miR-29b2* stem. Although the sequences of the two *pre-miR-29b* stems are differing, mature *miR-29b* resulting from these two stem structures is identical.

Consequently, we first investigated the transcriptional effects of CEBPA on the two *miR-29* loci to define the individual contribution of each locus to *miR-29b* expression. Our microarray data indicated a roughly two-fold induction of *miR-29a* expression after restoring CEBPA in Kasumi-1 cells, whereas *miR-29c* tended to be suppressed (−1.3-fold). By qRT-PCR, we confirmed that *miR-29a* was induced two-fold 72 h after CEBPA restoration, similarly to *miR-29b*. In contrast, the expression of *miR-29c* was not affected (Figure 3B). This suggests that *miR-29a* and *miR-29b*, but not *miR-29c*, are regulated by CEBPA.

Previous reports (Chang *et al*, 2008; Wang *et al*, 2008) indicated that mature *miR-29* family members encoded on the same chromosome are processed from a common primary transcript (pri-miRNA, Figure 3C). We thus designed a series of primer pairs dispersed over the *pri-miR-29a/b1* and *pri-miR-29b2/c* sequences. Again, we observed a two-fold induction of *pri-miR-29a/b1* (Figure 3D, left), whereas the expression of the *miR-29b2/c* primary transcript remained stable (Figure 3D, right). This is consistent with the above-mentioned observation of suppressed *miR-29b* in AML patients with aberrant chromosome 7q. We thus concluded that CEBPA activates the expression of the *miR-29a/b1* cluster on chromosome 7, whereas it does not affect *miR-29b2/c* on chromosome 1 in myeloid leukaemic cells.

### CEBPA specifically activates the *pri-miR-29a/b1* promoter

As previously shown by RACE experiments (Chang *et al*, 2008), the primary *miR-29a/b1* transcript starts 35.7 kb upstream of the *pre-miR-29b1* stem structure, and the highly conserved promoter region just upstream of this transcription start is responsible for regulation of *miR-29a/b1* expression (Figure 3C).

**Figure 4:**
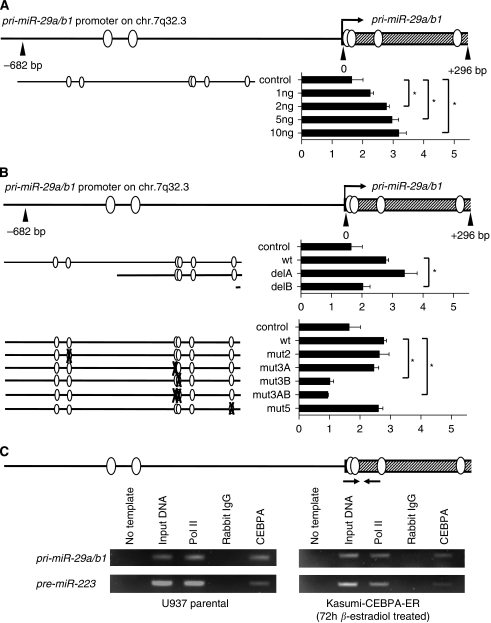
CEBPA activates the *pri-miR29a/b1* promoter on chromosome 7q32.3. Results represent three independant experiments and are given as fold changes compared with the empty pcDNA3 expression vector; ^*^P<0.05, control: empty pGL3b luciferase vector. (**A**) A computational analysis of the highly conserved region spanning −682 to +296 bp across the *pri-miR-29a/b1* transcription start site resulted in six putative CEBPA binding sites. This sequence was cloned into the pGL3b luciferase vector and 200 ng of promoter construct were transfected with 1–10 ng of *CEBPA* expression plasmid. (**B**) The CEBPA binding site +15 to +29 bp downstream of the *pri-miR-29a/b1* transcription start was identified to be responsible for *pri-miR-29a/b1* activation. Deleted (**B**, upper panel) or mutated (**B**, lower panel) *pri-miR-29a/b1* promoter construct (200 ng) were transfected with 2 ng *CEBPA* expression plasmid. (**C**) Chromatin immunoprecipitation (ChIP) assays confirmed binding of CEBPA to the site in the *pri-miR-29a/b1* transcription start region. Endogenous CEBPA of U937 cells (**C**, left) and exogenous CEBPA in Kasumi-1-CEBPA-ER cells (**C**, right) were binding to the CEBPA site identified by the luciferase assays above. Binding of CEBPA to the regulatory element of *pre-miR-223* was used as positive control for these ChIP experiments.

A computational analysis of the conserved region spanning −682 bp upstream to +296 bp downstream of the *pri-miR-29a/b1* transcription start site indicated six potential CEBP binding sites (Figure 4A). Using luciferase reporter assays, we observed that CEBPA, indeed, activated the entire conserved promoter region two-fold in a dose-dependent manner (Figure 4A). Deletion and mutation constructs of the *pri-miR-29a/b1* promoter identified a CEBP binding site located +15 to +29 bp immediately downstream of the transcription start site to be responsible for CEBPA-mediated activation of the *pri-miR-29a/b1* promoter (Figure 4B). Chromatin immunoprecipitation assays in myeloid leukaemic cells confirmed that CEBPA is, in fact, binding *in vivo* to this part of the *pri-miR-29a/b1* locus as suggested by the luciferase experiments (Figure 4C): both endogenous CEBPA in U937 cells and exogenous CEBPA in Kasumi-CEBPA-ER were binding to the CEBPA site located +15 to +29 bp downstream of the *pri-miR-29a/b1* transcript start.

Furthermore, we sought to exclude additional functional CEBPA-binding sites in the non-conserved region directly upstream of the *pre-miR29a* and *-29b1* stem structures. Such additional CEBPA responsive promoter elements were reported for CEBPA regulation of *miR-223* (Fazi *et al*, 2005; Eyholzer *et al*, 2009) or *miR-661* (Reddy *et al*, 2009). Although the computational sequence analysis of 2.2 kb upstream of the *pre-miR-29b1* stem indicated four putative CEBP binding sites, we found that none of them was CEBPA responsive in luciferase assays (data not shown). We thus conclude that CEBPA activates *miR-29a/b* expression through direct binding to a single site in the conserved promoter region of the *pri-miR-29a/b1* transcript on chromosome 7q32.3.

## Discussion

The transcription factor CEBPA is a master regulator within normal haematopoiesis (Pabst and Mueller, 2007; Koschmieder *et al*, 2009). Increasing evidence indicates that CEBPA is exerting its regulatory effects, at least in part, by direct regulation of specific miRNAs. Fazi *et al* (2005) first identified *miR-223* as a direct target of CEBPA. The activation of *miR-223* by CEBPA triggers granulocytic differentiation and maturation (Fazi *et al*, 2005, 2007). Recently, *miR-661* was reported to be another direct CEBPA target miRNA. *miR-661* suppresses the metastatic tumour antigen 1, a gene broadly upregulated in human cancer (Reddy *et al*, 2009).

In this study, we sought to identify the pattern of miRNAs that are regulated by CEBPA in haematopoietic cells. Using leukaemic Kasumi-1 cells with conditionally inducible CEBPA function (Pabst *et al*, 2001a), we determined the expression changes of 470 human miRNAs. We identified 18 miRNAs, whose expression levels were changed more than two-fold after restoring CEBPA function: *miR-98*, *miR-181b*, *miR-197*, *miR-210*, *miR-342*, *miR-432*, *miR-550*, and *miR-776* were suppressed, whereas *miR-29b*, *miR-223*, *miR-370*, *miR-496*, *miR-572*, *miR-575*, *miR-630*, *miR-638*, *miR-663*, and *miR-765* were induced compared with their expression levels before CEBPA induction. As expected, the previously identified CEBPA target *miR-223* was induced more than two-fold. In contrast, *miR-661* levels remained stable in our system of myeloid leukaemic cells. As *miR-661* activation by CEBPA was reported to be involved in some solid tumours (Reddy *et al*, 2009), it may be less important in haematopoietic cells.

So far, two studies described miRNA expression patterns associated with AML patients with *CEBPA* mutations (Jongen-Lavrencic *et al*, 2008; Marcucci *et al*, 2008). In accordance with our observations, *miR-181* family members were induced in patients with *CEBPA* mutations in both studies. Remarkably, however, these authors have not reported other miRNAs detected by our array approach. Possible explanations might be differences in the type of arrays used or differences arising from a comparison of results obtained from CEBPA restoration in a leukaemic cell line (as in this report) *vs* differing miRNA patterns seen in patients with or without *CEBPA* mutations.

Among the 18 identified miRNAs in our approach, we decided to dissect the molecular mechanisms involved in CEBPA-dependent regulation of *miR-29b* based on its increasingly recognised importance for normal haematopoiesis and leukaemogensis. Suppressed *miR-29* levels have been shown to be associated with disease progression in chronic lymphoid leukemia patients (Calin *et al*, 2005; Stamatopoulos *et al*, 2009; Visone *et al*, 2009). In AML, *miR-29* suppression is associated to translocations involving the *MLL* oncogene (Li *et al*, 2008), but it is induced in patients with *NPM1* mutations in the absence of *FLT3-ITD* alterations (Garzon *et al*, 2008). Consistent with these expression data suggesting tumour-suppressive properties, *miR-29* is reported to trigger differentiation (Wang *et al*, 2008; Garzon *et al*, 2009b; Li *et al*, 2009) and apoptosis (Mott *et al*, 2007; Garzon *et al*, 2009a; Park *et al*, 2009; Xiong *et al*, 2009) in various tissues as well as having anti-invasive and anti-proliferative properties in solid tumours (Muniyappa *et al*, 2009; Xiong *et al*, 2009).

Several potential oncogenes have been reported to be silenced by *miR-29*, such as *Tcl1* (Pekarsky *et al*, 2006), *YY1* (Wang *et al*, 2008), *CXXC6*, and *CDK6* (Garzon *et al*, 2009a), the p53 upstream inhibitors *p85α* and *CDC42* (Park *et al*, 2009), and the anti-apoptotic Bcl2 family members *Bcl2* and *Mcl1* (Mott *et al*, 2007; Garzon *et al*, 2009a; Xiong *et al*, 2009). Importantly, *miR-29* family members were also reported to have an important role in preventing epigenetic silencing of tumour suppressors due to *de novo* methylation in cancer, as they directly suppress DNMT3A and B (Fabbri *et al*, 2007; Garzon *et al*, 2009b).

Despite the variety of reported *miR-29* downstream effects, little is known so far on how *miR-29* expression is regulated itself. Chang *et al* (2008) first described the conserved promoter regions of both *miR-29* family clusters on chromosome 1q32.2 (*miR-29b2/c*) and chromosome 7q32.3 (*miR-29a/b1*). They showed that both clusters were suppressed by the oncogenic transcription factor Myc in B-cell lymphoma. Wang *et al* (2008) proposed that the *miR-29b2/c* cluster on chromosome 1 is suppressed in rhabdomyosarcoma through NFκB/YY1 via the same conserved upstream promoter region.

In this study, we report that the haematopoietic master transcription factor CEBPA is inducing *miR-29b* expression. We observed that *miR-29b* is suppressed in AML patients with disrupted CEBPA function. This comprises AML patients with *CEBPA* mutations or with suppressed CEBPA function because of t(8,21) or inv(16) chromosome aberrations (Pabst *et al*, 2001a, 2001b; Helbling *et al*, 2005). We also confirmed recent observations by others (Garzon *et al*, 2009a) that *miR-29b* is suppressed in AML patients with alterations of chromosome 7 (monosomy 7 or del(7q)). Interestingly, functional analysis of CEBPA-mediated *miR-29b* expression indicated that only the *miR-29a/b1* locus on chromosome 7q32.3 is activated by CEBPA, whereas *miR-29b2/c* expression is not affected by CEBPA. This was surprising as the conserved promoter region upstream of the *miR-29b2/c* primary transcript on chromosome 1q32.2 (Chang *et al*, 2008) also indicated several putative CEBPA binding sites in a computational analysis. However, we found that none of them turned out to be functional. The finding that CEBPA induces *miR-29b* expression only from its chromosome 7q32.3 locus (*miR-29a/b1* cluster) provides a rationale for *miR-29b* suppression observed in patients with alterations of chromosome 7.

In summary, using miRNA microarrays, we found that CEBPA affects the expression of a defined subset of 18 miRNAs in human AML cells. Among them, we identified the *miR-29a/b1* cluster encoded on chromosome 7q32.3 to be directly activated by CEBPA. The findings of our study suggest a rationale for *miR-29b* suppression in AML patients with disrupted CEBPA function or with aberrations on chromosome 7.

## Figures and Tables

**Table 1:** miRNAs affected by CEBPA in hematopoietic cells

			**Literature available**			
**miRNA**	**Chr. locus**	**Fold change** a	**Expression** b	**Regulation** c	**Targets** d	**Function** e
*(A) miRNAs* > *two-fold decreased after CEBPA restoration*						
*miR-98*	Xp11.2	−2.15	↑ Breast, lung cancer (Yan *et al*, 2008; Bhat-Nakshatri *et al*, 2009; Du *et al*, 2009)	↑ After Myc inhibition (Sampson *et al*, 2007)	*FUS1*, *E2F2*, *Myc*, *MGA2* (Hebert *et al*, 2007; Sampson *et al*, 2007; Bhat-Nakshatri *et al*, 2009; Du *et al*, 2009)	Potentiates doxorubicin, cisplatin resistance (Hebert *et al*, 2007)
*miR-181b miR-181* family	1q32.1 9q33.3	−2.40	↑ Erythropoiesis (Choong *et al*, 2007), B-cell differentiation (Chen *et al*, 2004); ↓ megakaryopoiesis (Garzon *et al*, 2006) ↑ AML with CEBPA mut (Marcucci *et al*, 2008); ↓ in ATRA or HNE differentiated leukemic cells (Garzon *et al*, 2007; Careccia *et al*, 2009; Pizzimenti *et al*, 2009), CLL (Pekarsky *et al*, 2006); ↓ associated to disease progression in CN-AML (Marcucci *et al*, 2008); ↑↓ various solid tumors (Miller *et al*, 2008; Wong *et al*, 2008; Yan *et al*, 2008; Conti *et al*, 2009; Ji *et al*, 2009)	↑ Hypoxia, by HIF1*α* (Kulshreshtha *et al*, 2007)	*TCL1* (Pekarsky *et al*, 2006); *GATA6, CDX2, NLK* (Ji *et al*, 2009)	↑ B-cell differentiation (Chen *et al*, 2004), ↓ differentiation in hepatocellular carcinoma (Ji *et al*, 2009); ↓ proliferation, invasion of glioma (Shi *et al*, 2008), lung cancer (Cheng *et al*, 2005), ↑ apoptosis (Shi *et al*, 2008)
*miR-197*	1p13.3	−2.26	↑ Various solid tumors (Weber *et al*, 2006; Wang *et al*, 2007; Nikiforova *et al*, 2008; Wong *et al*, 2008; Du *et al*, 2009).		*FUS1* (Du *et al*, 2009), *ACVR1, TSPAN3* (Weber *et al*, 2006)	↑ Proliferation (Weber *et al*, 2006)
*miR-210*	11p15.5	−2.13	↑ Erythroid differentiation (Bianchi *et al*, 2009); ↑ in various solid tumors and other diseases (Camps *et al*, 2008; Foekens *et al*, 2008; Bimpaki *et al*, 2009; Cho *et al*, 2009; Malzkorn *et al*, 2009; Satzger *et al*, 2009; Tombol *et al*, 2009; Jung *et al*, 2009; Greither *et al*, 2010)	↑ Hypoxia (Kulshreshtha *et al*, 2007; Giannakakis *et al*, 2008; Pulkkinen *et al*, 2008), by HIF1*α* (Camps *et al*, 2008; Pulkkinen *et al*, 2008)	*E2F3* (Giannakakis *et al*, 2008), *MNT* (Zhang *et al*, 2009), *RAD52* (Crosby *et al*, 2009), (Fasanaro *et al*, 2008)	↑ Cell proliferation (Zhang *et al*, 2009), angiogenesis, cell migration (Fasanaro *et al*, 2008); ↓ Pro-apoptotic signaling (Kulshreshtha *et al*, 2007), DNA repair (Crosby *et al*, 2009); potential biomarker: detectable in plasma/sera of pancreatic adenocarinoma (Wang *et al*, 2009), lymphoma patients (Lawrie *et al*, 2008)
*miR-342*	14q32.2	−2.16	↑ After ATRA differentiation (Garzon *et al*, 2007; Careccia *et al*, 2009; De Marchis *et al*, 2009), ↑↓ Hematologic diseases (Guglielmelli *et al*, 2007; Ronchetti *et al*, 2008), solid tumors (Grady *et al*, 2008; Miller *et al*, 2008; Lowery *et al*, 2009)	↑ By PU.1, IRF-9 (De Marchis *et al*, 2009), ↓ by PML/RARA, IRF-1(Careccia *et al*, 2009; De Marchis *et al*, 2009)		↑ Differentiation (De Marchis *et al*, 2009) apoptosis (Grady *et al*, 2008)
*miR-432*	14q32.3	−2.20				
*miR-550*	7p15.1	−2.18				
*miR-766*	Xq24	−2.59				
						
*(B) miRNAs* > *two-fold increased after CEBPA restoration*						
*miR-29b miR-29* family	1q32.2 7q32.3	+2.03	↓ AML in general (Garzon *et al*, 2008), patients with NPM1 mut + lack FLT3-ITD (Garzon *et al*, 2008), MLL translocations (Garzon *et al*, 2008; Li *et al*, 2008), -7q (Garzon *et al*, 2009); CLL disease progression (Calin *et al*, 2005; Stamatopoulos *et al*, 2009; Visone *et al*, 2009); various solid tumors (Fabbri *et al*, 2007; Mott *et al*, 2007; Wang *et al*, 2008; Xiong *et al*, 2009)	↓ By Myc (Chang *et al*, 2008), NF*κ*B, YY1 (Wang *et al*, 2008)	*p85α, CDC42* (Park *et al*, 2009); *Bcl-2, Mcl-1*(Mott *et al*, 2007; Garzon *et al*, 2009; Xiong *et al*, 2009); *Tcl1* (Pekarsky *et al*, 2006), *YY1* (Wang *et al*, 2008), *CXXC6, CDK6* (Garzon *et al*, 2009) *DNMT3A,B* (Fabbri *et al*, 2007; Garzon *et al*, 2009)	↑ Differentiation (Wang *et al*, 2008; Garzon *et al*, 2009; Li *et al*, 2009) apoptosis (Mott *et al*, 2007; Garzon *et al*, 2009; Park *et al*, 2009; Xiong *et al*, 2009); ↓ proliferation (Muniyappa *et al*, 2009; Xiong *et al*, 2009), cell migration (Muniyappa *et al*, 2009), *de novo* methylation (Fabbri *et al*, 2007; Garzon *et al*, 2009)
*miR-223*	Xq12	+2.02	↑ normal myeloid cells (Chen *et al*, 2004; Ramkissoon *et al*, 2006; Johnnidis *et al*, 2008); ↓ various leukemia types (Debernardi *et al*, 2007; Mi *et al*, 2007; Stamatopoulos *et al*, 2009)	↑ By CEBPA/B (Fazi *et al*, 2005; Fukao *et al*, 2007; Eyholzer *et al*, 2009), ↓ by NFIA (Fazi *et al*, 2005), AML1-ETO (Fazi *et al*, 2007)	*E2F1*(Pulikkan *et al*, 2009), *NFIA* (Fazi *et al*, 2005), *LMO2* (Felli *et al*, 2009)	↑ Differentiation (Fazi *et al*, 2005; Fazi *et al*, 2007), maturation, functionality of granulocytes (Johnnidis *et al*, 2008); ↓ erythroid differentiation (Felli *et al*, 2009); cell cycle progression (Pulikkan *et al*, 2009)
*miR-370*	14q32.3	+3.14	↑ AML with t(15;17) (Dixon-McIver *et al*, 2008),↓ solid tumors, progression (Haller *et al*, 2010; Meng *et al*, 2008)	↓ By IL-7 (Meng *et al*, 2008)	*MAP3K8* (Meng *et al*, 2008)	↓ Growth of malignant cholangiocytes (Meng *et al*, 2008)
*miR-496*	14q32.3	+4.30				
*miR-572*	4p15.3	+3.00				
*miR-575*	4q21.2	+2.82	↓ in HNE differentiated HL60 leukemic cells (Pizzimenti *et al*, 2009)			
*miR-630*	15q24.1	+4.73				
*miR-638*	19p13.2	+2.12	↑ in senescent fibroblasts (Maes *et al*, 2009); ↓ ratio miR-92/-638 in AML, ALL (Tanaka *et al*, 2009)			
*miR-663*	20p11.1	+3.18	↑ in HNE differentiated HL60 leukemic cells (Pizzimenti *et al*, 2009), senescent fibroblasts (Maes *et al*, 2009); hypermethylated in breast cancer (Lehmann *et al*, 2008)			
*miR-765*	1q23.1	+2.02				

Abbreviations: AML=acute myeloid leukemia; CEBPA=CCAAT/enhancer-binding protein-*α*; CLL=chronic lymphoid leukemia; IL=interleukin; miRNA=microRNA; NF *κ*B=nuclear factor *κ*B; NFIA=nuclear factor IA.

References are given in Supplementary Table 3.

a Mean expression change (suppression (−) or induction (+)) out of two miRNA microarray experiments, analyzed with GeneSpring GX 9.0 software.

b Expression data summarized with ↑ for induction or ↓ suppression of miRNA referenced in literature. For miRNAs with differing expression depending on tissue context, expression is shown as ↑↓.

c Direct activator ↑ or suppressor ↓ of referenced miRNA.

d Functionally tested and verified targets only.

e Effect of miRNA: on cellular mechanisms induced ↑ or suppressed ↓ by a particular miRNA.

## Supplementary Material

Supplementary Table S2

References to Table 1
