# Supplementary material for: The tumour-suppressive miR-29a/b1 cluster is regulated by CEBPA and blocked in human AML
Source: Br J Cancer. 2010 Jul 13;103(2):275–84. doi: 10.1038/sj.bjc.6605751 (PMC2906742; doi:10.1038/sj.bjc.6605751)
Supplement: Supplementary Table S1 [file 6605751x1.doc]

**Supplementary table S1.** Characteristics of AML patients and healthy volunteers

| AML patients |  |  |  |  |  |  |  |  |
| --- | --- | --- | --- | --- | --- | --- | --- | --- |
|  |  |  |  |  |  |  |  | normal |
| FAB classification | n | median age; range | *CEBPA* mut | t(8;21) | inv(16) | -7q | others[[1]](#footnote-2)* | karyotype[[2]](#footnote-3)‡ |
| M0 | 9 | 60; 24-75 |  |  |  | 1 | 4 | 4 |
| M1 | 16 | 60; 41-80 | 2 |  |  | 3 | 6 | 7 |
| M2 | 16 | 60; 20-81 | 4 | 6 |  |  | 2 | 8 |
| M3 | 5 | 57; 35-77 |  |  |  |  | 5 | 0 |
| M4 | 11 | 47; 37-79 |  |  | 3 | 2 | 0 | 6 |
| M5 | 7 | 60; 32-78 |  |  |  | 1 | 0 | 6 |
| M6 | 1 | 51 |  |  |  |  | 0 | 1 |
| M7 | 1 | 39 |  |  |  |  | 0 | 1 |
| total | 66 | 9.5; 20-81 | 6 | 6 | 3 | 7 | 17 | 33 |
| CD34+ samples | 3 | 45; 43-51 |  |  |  |  |  |  |
| healthy volunteers | 6 | 41; 28-72 |  |  |  |  |  |  |

1. ^^ other chromosomal alterations: 5 M3 patients (pts) with t(15;17); 7 pts complex (>two chromosomal alterations); 1 pt (–Y); 1 pt t(6;9); 1 pt t(2;11);

   1 pt trisomy 11; 1 pt tetrasomy 21. [↑](#footnote-ref-2)
2. ^‡^ including also all *CEBPA* mut patients. [↑](#footnote-ref-3)
