# Supplementary material for: The tumour-suppressive miR-29a/b1 cluster is regulated by CEBPA and blocked in human AML
Source: Br J Cancer. 2010 Jul 13;103(2):275–84. doi: 10.1038/sj.bjc.6605751 (PMC2906742; doi:10.1038/sj.bjc.6605751)
Supplement: Supplementary Table S2 [file 6605751x2.doc]

**Supplementary table S2. Oligonucleotides**

| Quantitative RT-PCR detection of *pri-miR-29b* transcripts | | | | |
| --- | --- | --- | --- | --- |
| *pri-miR-29a/b1*  (chr. 7q32.3) | exon 1 | | fw  rev | 5’-TAC TGA ACT GTC ACG GCA GA-3’  5’-TGT AGT TAG CGA CCT CTG CT-3’ |
|  | exon 4 | | fw  rev | 5’-TTG CAC CCT CAC GAC ATG CT-3’  5’-TGA CTC TCA GCA GGC CTC A-3’ |
| *pri-miR-29b2/c*  (chr. 1q32.2) | exon 1 | | fw  rev | 5’-ACT TCT TTA GGG GTG TGC GTA-3’  5’-ACC CAT CTC CCT AGC ATT CT-3’ |
|  | exon 6 | | fw  rev | 5’-TCA GAC TTG CCA CCT GGA CT-3’  5’-AGT TGG CAT GAG GCT TCG A-3’ |
| Cloning of *pri-miR-29a/b1* promoter constructs | | | | |
| wild type  (-682bp to +296bp) | fw  rev | 5’-AGG GTT AAT GCT CCA ACC-3’  5’-TGT AGT TAG CGA CCT CTG CT-3’ | | |
| deletion A  (KpnI RE site mut) | wt  mut | 5’-CCG CCC AGA CGG ATC TGC AGA CTG GCC CC-3’  5’-CCG CCC AGA CGG ***TA***C ***C***GC AGA CTG GCC CC-3’ | | |
| deletion B |  | cut at mutation 5 (KpnI restriction site) | | |
| mutation 2 | wt  mut | 5’-CAC AGG GGT TGA GAA ACC AAA TTA AAA TTA G-3’  5’-CAC AGG GGT TGA G***GT*** ACC AAA TTA AAA TTA G-3’ | | |
| mutation 3A | wt  mut | 5’-GCC ACA GTT TTT TGG AAT GAA CGT TGT GAA ATC-3’  5’-GCC ACA GTT TTT TGG ***T***A***C*** CAA CGT TGT GAA ATC-3’ | | |
| mutation 3B | wt  mut | 5’-ATG AAC GTT GTG AAA TCC CTC CTT TAT AAT G-3’  5’-ATG AAC GTT GTG ***GT***A ***C***CC CTC CTT TAT AAT G-3’ | | |
| mutation 5 | wt  mut | 5’-CAG GAG CTG GTG ATT TCC TAA GCA GAG GTC-3’  5’-CAG GAG CTG GTG ***GG***T***A***CC TAA GCA GAG GTC-3’ | | |
|  |  |  | | |
| Chromatin immunoprecipitation assay (ChIP) | | | | |
| *pri-miR-29a/b1* promoter | | fw  rev | 5’-ACT TCT CAG AGG GCA GGC T-3’  5’-TCT GAT AAA ACC ACC AAC TGA-3’ | |
| *pre-miR-223* promoter  (Fazi et al, 2005) | | fw  rev | 5’-GCC CTC TTT GTT GAT GTG TC-3’  5’-GGC AGC TAT TAA AGT GCC CT-3’ | |
