# Supplementary material for: The tumour-suppressive miR-29a/b1 cluster is regulated by CEBPA and blocked in human AML
Source: Br J Cancer. 2010 Jul 13;103(2):275–84. doi: 10.1038/sj.bjc.6605751 (PMC2906742; doi:10.1038/sj.bjc.6605751)
Supplement: References to Table 1 [file 6605751x3.doc]

**Supplementary table S3.**  References to table 1

1. Yan, L. X., Huang, X. F., Shao, Q., Huang, M. Y., Deng, L., Wu, Q. L., Zeng, Y. X., and Shao, J. Y. MicroRNA miR-21 overexpression in human breast cancer is associated with advanced clinical stage, lymph node metastasis and patient poor prognosis. Rna, *14:* 2348-2360, 2008.

2. Bhat-Nakshatri, P., Wang, G., Collins, N. R., Thomson, M. J., Geistlinger, T. R., Carroll, J. S., Brown, M., Hammond, S., Srour, E. F., Liu, Y., and Nakshatri, H. Estradiol-regulated microRNAs control estradiol response in breast cancer cells. Nucleic Acids Res, *37:* 4850-4861, 2009.

3. Du, L., Schageman, J. J., Subauste, M. C., Saber, B., Hammond, S. M., Prudkin, L., Wistuba, II, Ji, L., Roth, J. A., Minna, J. D., and Pertsemlidis, A. miR-93, miR-98, and miR-197 regulate expression of tumor suppressor gene FUS1. Mol Cancer Res, *7:* 1234-1243, 2009.

4. Sampson, V. B., Rong, N. H., Han, J., Yang, Q., Aris, V., Soteropoulos, P., Petrelli, N. J., Dunn, S. P., and Krueger, L. J. MicroRNA let-7a down-regulates MYC and reverts MYC-induced growth in Burkitt lymphoma cells. Cancer Res, *67:* 9762-9770, 2007.

5. Hebert, C., Norris, K., Scheper, M. A., Nikitakis, N., and Sauk, J. J. High mobility group A2 is a target for miRNA-98 in head and neck squamous cell carcinoma. Mol Cancer, *6:* 5, 2007.

6. Choong, M. L., Yang, H. H., and McNiece, I. MicroRNA expression profiling during human cord blood-derived CD34 cell erythropoiesis. Exp Hematol, *35:* 551-564, 2007.

7. Chen, C. Z., Li, L., Lodish, H. F., and Bartel, D. P. MicroRNAs modulate hematopoietic lineage differentiation. Science, *303:* 83-86, 2004.

8. Garzon, R., Pichiorri, F., Palumbo, T., Iuliano, R., Cimmino, A., Aqeilan, R., Volinia, S., Bhatt, D., Alder, H., Marcucci, G., Calin, G. A., Liu, C. G., Bloomfield, C. D., Andreeff, M., and Croce, C. M. MicroRNA fingerprints during human megakaryocytopoiesis. Proc Natl Acad Sci U S A, *103:* 5078-5083, 2006.

9. Marcucci, G., Maharry, K., Radmacher, M. D., Mrozek, K., Vukosavljevic, T., Paschka, P., Whitman, S. P., Langer, C., Baldus, C. D., Liu, C. G., Ruppert, A. S., Powell, B. L., Carroll, A. J., Caligiuri, M. A., Kolitz, J. E., Larson, R. A., and Bloomfield, C. D. Prognostic significance of, and gene and microRNA expression signatures associated with, CEBPA mutations in cytogenetically normal acute myeloid leukemia with high-risk molecular features: a Cancer and Leukemia Group B Study. J Clin Oncol, *26:* 5078-5087, 2008.

10. Marcucci, G., Radmacher, M. D., Maharry, K., Mrozek, K., Ruppert, A. S., Paschka, P., Vukosavljevic, T., Whitman, S. P., Baldus, C. D., Langer, C., Liu, C. G., Carroll, A. J., Powell, B. L., Garzon, R., Croce, C. M., Kolitz, J. E., Caligiuri, M. A., Larson, R. A., and Bloomfield, C. D. MicroRNA expression in cytogenetically normal acute myeloid leukemia. N Engl J Med, *358:* 1919-1928, 2008.

11. Garzon, R., Pichiorri, F., Palumbo, T., Visentini, M., Aqeilan, R., Cimmino, A., Wang, H., Sun, H., Volinia, S., Alder, H., Calin, G. A., Liu, C. G., Andreeff, M., and Croce, C. M. MicroRNA gene expression during retinoic acid-induced differentiation of human acute promyelocytic leukemia. Oncogene, *26:* 4148-4157, 2007.

12. Careccia, S., Mainardi, S., Pelosi, A., Gurtner, A., Diverio, D., Riccioni, R., Testa, U., Pelosi, E., Piaggio, G., Sacchi, A., Lavorgna, S., Lo-Coco, F., Blandino, G., Levrero, M., and Rizzo, M. G. A restricted signature of miRNAs distinguishes APL blasts from normal promyelocytes. Oncogene, *28:* 4034-4040, 2009.

13. Pizzimenti, S., Ferracin, M., Sabbioni, S., Toaldo, C., Pettazzoni, P., Dianzani, M. U., Negrini, M., and Barrera, G. MicroRNA expression changes during human leukemic HL-60 cell differentiation induced by 4-hydroxynonenal, a product of lipid peroxidation. Free Radic Biol Med, *46:* 282-288, 2009.

14. Pekarsky, Y., Santanam, U., Cimmino, A., Palamarchuk, A., Efanov, A., Maximov, V., Volinia, S., Alder, H., Liu, C. G., Rassenti, L., Calin, G. A., Hagan, J. P., Kipps, T., and Croce, C. M. Tcl1 expression in chronic lymphocytic leukemia is regulated by miR-29 and miR-181. Cancer Res, *66:* 11590-11593, 2006.

15. Miller, T. E., Ghoshal, K., Ramaswamy, B., Roy, S., Datta, J., Shapiro, C. L., Jacob, S., and Majumder, S. MicroRNA-221/222 confers tamoxifen resistance in breast cancer by targeting p27Kip1. J Biol Chem, *283:* 29897-29903, 2008.

16. Wong, T. S., Liu, X. B., Wong, B. Y., Ng, R. W., Yuen, A. P., and Wei, W. I. Mature miR-184 as Potential Oncogenic microRNA of Squamous Cell Carcinoma of Tongue. Clin Cancer Res, *14:* 2588-2592, 2008.

17. Ji, J., Yamashita, T., Budhu, A., Forgues, M., Jia, H. L., Li, C., Deng, C., Wauthier, E., Reid, L. M., Ye, Q. H., Qin, L. X., Yang, W., Wang, H. Y., Tang, Z. Y., Croce, C. M., and Wang, X. W. Identification of microRNA-181 by genome-wide screening as a critical player in EpCAM-positive hepatic cancer stem cells. Hepatology, *50:* 472-480, 2009.

18. Conti, A., Aguennouz, M., La Torre, D., Tomasello, C., Cardali, S., Angileri, F. F., Maio, F., Cama, A., Germano, A., Vita, G., and Tomasello, F. miR-21 and 221 upregulation and miR-181b downregulation in human grade II-IV astrocytic tumors. J Neurooncol, *93:* 325-332, 2009.

19. Kulshreshtha, R., Ferracin, M., Wojcik, S. E., Garzon, R., Alder, H., Agosto-Perez, F. J., Davuluri, R., Liu, C. G., Croce, C. M., Negrini, M., Calin, G. A., and Ivan, M. A microRNA signature of hypoxia. Mol Cell Biol, *27:* 1859-1867, 2007.

20. Shi, L., Cheng, Z., Zhang, J., Li, R., Zhao, P., Fu, Z., and You, Y. hsa-mir-181a and hsa-mir-181b function as tumor suppressors in human glioma cells. Brain Res, *1236:* 185-193, 2008.

21. Cheng, A. M., Byrom, M. W., Shelton, J., and Ford, L. P. Antisense inhibition of human miRNAs and indications for an involvement of miRNA in cell growth and apoptosis. Nucleic Acids Res, *33:* 1290-1297, 2005.

22. Weber, F., Teresi, R. E., Broelsch, C. E., Frilling, A., and Eng, C. A limited set of human MicroRNA is deregulated in follicular thyroid carcinoma. J Clin Endocrinol Metab, *91:* 3584-3591, 2006.

23. Nikiforova, M. N., Tseng, G. C., Steward, D., Diorio, D., and Nikiforov, Y. E. MicroRNA expression profiling of thyroid tumors: biological significance and diagnostic utility. J Clin Endocrinol Metab, *93:* 1600-1608, 2008.

24. Wang, T., Zhang, X., Obijuru, L., Laser, J., Aris, V., Lee, P., Mittal, K., Soteropoulos, P., and Wei, J. J. A micro-RNA signature associated with race, tumor size, and target gene activity in human uterine leiomyomas. Genes Chromosomes Cancer, *46:* 336-347, 2007.

25. Bianchi, N., Zuccato, C., Lampronti, I., Borgatti, M., and Gambari, R. Expression of miR-210 during erythroid differentiation and induction of gamma-globin gene expression. BMB Rep, *42:* 493-499, 2009.

26. Camps, C., Buffa, F. M., Colella, S., Moore, J., Sotiriou, C., Sheldon, H., Harris, A. L., Gleadle, J. M., and Ragoussis, J. hsa-miR-210 Is induced by hypoxia and is an independent prognostic factor in breast cancer. Clin Cancer Res, *14:* 1340-1348, 2008.

27. Foekens, J. A., Sieuwerts, A. M., Smid, M., Look, M. P., de Weerd, V., Boersma, A. W., Klijn, J. G., Wiemer, E. A., and Martens, J. W. Four miRNAs associated with aggressiveness of lymph node-negative, estrogen receptor-positive human breast cancer. Proc Natl Acad Sci U S A, *105:* 13021-13026, 2008.

28. Cho, W. C., Chow, A. S., and Au, J. S. Restoration of tumour suppressor hsa-miR-145 inhibits cancer cell growth in lung adenocarcinoma patients with epidermal growth factor receptor mutation. Eur J Cancer, *45:* 2197-2206, 2009.

29. Jung, M., Mollenkopf, H. J., Grimm, C., Wagner, I., Albrecht, M., Waller, T., Pilarsky, C., Johannsen, M., Stephan, C., Lehrach, H., Nietfeld, W., Rudel, T., Jung, K., and Kristiansen, G. MicroRNA profiling of clear cell renal cell cancer identifies a robust signature to define renal malignancy. J Cell Mol Med, 2009.

30. Greither, T., Grochola, L. F., Udelnow, A., Lautenschlager, C., Wurl, P., and Taubert, H. Elevated expression of microRNAs 155, 203, 210 and 222 in pancreatic tumors is associated with poorer survival. Int J Cancer, *126:* 73-80.

31. Tombol, Z., Szabo, P. M., Molnar, V., Wiener, Z., Tolgyesi, G., Horanyi, J., Riesz, P., Reismann, P., Patocs, A., Liko, I., Gaillard, R. C., Falus, A., Racz, K., and Igaz, P. Integrative molecular bioinformatics study of human adrenocortical tumors: microRNA, tissue-specific target prediction, and pathway analysis. Endocr Relat Cancer, *16:* 895-906, 2009.

32. Bimpaki, E. I., Iliopoulos, D., Moraitis, A., and Stratakis, C. A. MicroRNA signature in massive macronodular adrenocortical disease and implications for adrenocortical tumorigenesis. Clin Endocrinol (Oxf), 2009.

33. Satzger, I., Mattern, A., Kuettler, U., Weinspach, D., Voelker, B., Kapp, A., and Gutzmer, R. MicroRNA-15b represents an independent prognostic parameter and is correlated with tumor cell proliferation and apoptosis in malignant melanoma. Int J Cancer, 2009.

34. Malzkorn, B., Wolter, M., Liesenberg, F., Grzendowski, M., Stuhler, K., Meyer, H. E., and Reifenberger, G. Identification and Functional Characterization of microRNAs Involved in the Malignant Progression of Gliomas. Brain Pathol, 2009.

35. Giannakakis, A., Sandaltzopoulos, R., Greshock, J., Liang, S., Huang, J., Hasegawa, K., Li, C., O'Brien-Jenkins, A., Katsaros, D., Weber, B. L., Simon, C., Coukos, G., and Zhang, L. miR-210 links hypoxia with cell cycle regulation and is deleted in human epithelial ovarian cancer. Cancer Biol Ther, *7:* 255-264, 2008.

36. Pulkkinen, K., Malm, T., Turunen, M., Koistinaho, J., and Yla-Herttuala, S. Hypoxia induces microRNA miR-210 in vitro and in vivo ephrin-A3 and neuronal pentraxin 1 are potentially regulated by miR-210. FEBS Lett, *582:* 2397-2401, 2008.

37. Zhang, Z., Sun, H., Dai, H., Walsh, R. M., Imakura, M., Schelter, J., Burchard, J., Dai, X., Chang, A. N., Diaz, R. L., Marszalek, J. R., Bartz, S. R., Carleton, M., Cleary, M. A., Linsley, P. S., and Grandori, C. MicroRNA miR-210 modulates cellular response to hypoxia through the MYC antagonist MNT. Cell Cycle, *8:* 2756-2768, 2009.

38. Crosby, M. E., Kulshreshtha, R., Ivan, M., and Glazer, P. M. MicroRNA regulation of DNA repair gene expression in hypoxic stress. Cancer Res, *69:* 1221-1229, 2009.

39. Fasanaro, P., D'Alessandra, Y., Di Stefano, V., Melchionna, R., Romani, S., Pompilio, G., Capogrossi, M. C., and Martelli, F. MicroRNA-210 modulates endothelial cell response to hypoxia and inhibits the receptor tyrosine kinase ligand Ephrin-A3. J Biol Chem, *283:* 15878-15883, 2008.

40. Wang, J., Chen, J., Chang, P., LeBlanc, A., Li, D., Abbruzzesse, J. L., Frazier, M. L., Killary, A. M., and Sen, S. MicroRNAs in plasma of pancreatic ductal adenocarcinoma patients as novel blood-based biomarkers of disease. Cancer Prev Res (Phila Pa), *2:* 807-813, 2009.

41. Lawrie, C. H., Gal, S., Dunlop, H. M., Pushkaran, B., Liggins, A. P., Pulford, K., Banham, A. H., Pezzella, F., Boultwood, J., Wainscoat, J. S., Hatton, C. S., and Harris, A. L. Detection of elevated levels of tumour-associated microRNAs in serum of patients with diffuse large B-cell lymphoma. Br J Haematol, *141:* 672-675, 2008.

42. De Marchis, M. L., Ballarino, M., Salvatori, B., Puzzolo, M. C., Bozzoni, I., and Fatica, A. A new molecular network comprising PU.1, interferon regulatory factor proteins and miR-342 stimulates ATRA-mediated granulocytic differentiation of acute promyelocytic leukemia cells. Leukemia, *23:* 856-862, 2009.

43. Guglielmelli, P., Tozzi, L., Pancrazzi, A., Bogani, C., Antonioli, E., Ponziani, V., Poli, G., Zini, R., Ferrari, S., Manfredini, R., Bosi, A., and Vannucchi, A. M. MicroRNA expression profile in granulocytes from primary myelofibrosis patients. Exp Hematol, *35:* 1708-1718, 2007.

44. Ronchetti, D., Lionetti, M., Mosca, L., Agnelli, L., Andronache, A., Fabris, S., Deliliers, G. L., and Neri, A. An integrative genomic approach reveals coordinated expression of intronic miR-335, miR-342, and miR-561 with deregulated host genes in multiple myeloma. BMC Med Genomics, *1:* 37, 2008.

45. Bruchova, H., Merkerova, M., and Prchal, J. T. Aberrant expression of microRNA in polycythemia vera. Haematologica, *93:* 1009-1016, 2008.

46. Lowery, A. J., Miller, N., Devaney, A., McNeill, R. E., Davoren, P. A., Lemetre, C., Benes, V., Schmidt, S., Blake, J., Ball, G., and Kerin, M. J. MicroRNA signatures predict oestrogen receptor, progesterone receptor and HER2/neu receptor status in breast cancer. Breast Cancer Res, *11:* R27, 2009.

47. Grady, W. M., Parkin, R. K., Mitchell, P. S., Lee, J. H., Kim, Y. H., Tsuchiya, K. D., Washington, M. K., Paraskeva, C., Willson, J. K., Kaz, A. M., Kroh, E. M., Allen, A., Fritz, B. R., Markowitz, S. D., and Tewari, M. Epigenetic silencing of the intronic microRNA hsa-miR-342 and its host gene EVL in colorectal cancer. Oncogene, *27:* 3880-3888, 2008.

48. Garzon, R., Volinia, S., Liu, C. G., Fernandez-Cymering, C., Palumbo, T., Pichiorri, F., Fabbri, M., Coombes, K., Alder, H., Nakamura, T., Flomenberg, N., Marcucci, G., Calin, G. A., Kornblau, S. M., Kantarjian, H., Bloomfield, C. D., Andreeff, M., and Croce, C. M. MicroRNA signatures associated with cytogenetics and prognosis in acute myeloid leukemia. Blood, *111:* 3183-3189, 2008.

49. Garzon, R., Garofalo, M., Martelli, M. P., Briesewitz, R., Wang, L., Fernandez-Cymering, C., Volinia, S., Liu, C. G., Schnittger, S., Haferlach, T., Liso, A., Diverio, D., Mancini, M., Meloni, G., Foa, R., Martelli, M. F., Mecucci, C., Croce, C. M., and Falini, B. Distinctive microRNA signature of acute myeloid leukemia bearing cytoplasmic mutated nucleophosmin. Proc Natl Acad Sci U S A, *105:* 3945-3950, 2008.

50. Li, Z., Lu, J., Sun, M., Mi, S., Zhang, H., Luo, R. T., Chen, P., Wang, Y., Yan, M., Qian, Z., Neilly, M. B., Jin, J., Zhang, Y., Bohlander, S. K., Zhang, D. E., Larson, R. A., Le Beau, M. M., Thirman, M. J., Golub, T. R., Rowley, J. D., and Chen, J. Distinct microRNA expression profiles in acute myeloid leukemia with common translocations. Proc Natl Acad Sci U S A, *105:* 15535-15540, 2008.

51. Garzon, R., Heaphy, C. E., Havelange, V., Fabbri, M., Volinia, S., Tsao, T., Zanesi, N., Kornblau, S. M., Marcucci, G., Calin, G. A., Andreeff, M., and Croce, C. M. MicroRNA 29b functions in acute myeloid leukemia. Blood, 2009.

52. Calin, G. A., Ferracin, M., Cimmino, A., Di Leva, G., Shimizu, M., Wojcik, S. E., Iorio, M. V., Visone, R., Sever, N. I., Fabbri, M., Iuliano, R., Palumbo, T., Pichiorri, F., Roldo, C., Garzon, R., Sevignani, C., Rassenti, L., Alder, H., Volinia, S., Liu, C. G., Kipps, T. J., Negrini, M., and Croce, C. M. A MicroRNA signature associated with prognosis and progression in chronic lymphocytic leukemia. N Engl J Med, *353:* 1793-1801, 2005.

53. Stamatopoulos, B., Meuleman, N., Haibe-Kains, B., Saussoy, P., Van Den Neste, E., Michaux, L., Heimann, P., Martiat, P., Bron, D., and Lagneaux, L. microRNA-29c and microRNA-223 down-regulation has in vivo significance in chronic lymphocytic leukemia and improves disease risk stratification. Blood, *113:* 5237-5245, 2009.

54. Visone, R., Rassenti, L. Z., Veronese, A., Taccioli, C., Costinean, S., Aguda, B. D., Volinia, S., Ferracin, M., Palatini, J., Balatti, V., Alder, H., Negrini, M., Kipps, T. J., and Croce, C. M. Karyotype-specific microRNA signature in chronic lymphocytic leukemia. Blood, *114:* 3872-3879, 2009.

55. Fabbri, M., Garzon, R., Cimmino, A., Liu, Z., Zanesi, N., Callegari, E., Liu, S., Alder, H., Costinean, S., Fernandez-Cymering, C., Volinia, S., Guler, G., Morrison, C. D., Chan, K. K., Marcucci, G., Calin, G. A., Huebner, K., and Croce, C. M. MicroRNA-29 family reverts aberrant methylation in lung cancer by targeting DNA methyltransferases 3A and 3B. Proc Natl Acad Sci U S A, *104:* 15805-15810, 2007.

56. Mott, J. L., Kobayashi, S., Bronk, S. F., and Gores, G. J. mir-29 regulates Mcl-1 protein expression and apoptosis. Oncogene, *26:* 6133-6140, 2007.

57. Wang, H., Garzon, R., Sun, H., Ladner, K. J., Singh, R., Dahlman, J., Cheng, A., Hall, B. M., Qualman, S. J., Chandler, D. S., Croce, C. M., and Guttridge, D. C. NF-kappaB-YY1-miR-29 regulatory circuitry in skeletal myogenesis and rhabdomyosarcoma. Cancer Cell, *14:* 369-381, 2008.

58. Xiong, Y., Fang, J. H., Yun, J. P., Yang, J., Zhang, Y., Jia, W. H., and Zhuang, S. M. Effects of MicroRNA-29 on apoptosis, tumorigenicity, and prognosis of hepatocellular carcinoma. Hepatology, 2009.

59. Chang, T. C., Yu, D., Lee, Y. S., Wentzel, E. A., Arking, D. E., West, K. M., Dang, C. V., Thomas-Tikhonenko, A., and Mendell, J. T. Widespread microRNA repression by Myc contributes to tumorigenesis. Nat Genet, *40:* 43-50, 2008.

60. Park, S. Y., Lee, J. H., Ha, M., Nam, J. W., and Kim, V. N. miR-29 miRNAs activate p53 by targeting p85 alpha and CDC42. Nat Struct Mol Biol, *16:* 23-29, 2009.

61. Garzon, R., Liu, S., Fabbri, M., Liu, Z., Heaphy, C. E., Callegari, E., Schwind, S., Pang, J., Yu, J., Muthusamy, N., Havelange, V., Volinia, S., Blum, W., Rush, L. J., Perrotti, D., Andreeff, M., Bloomfield, C. D., Byrd, J. C., Chan, K., Wu, L. C., Croce, C. M., and Marcucci, G. MicroRNA-29b induces global DNA hypomethylation and tumor suppressor gene reexpression in acute myeloid leukemia by targeting directly DNMT3A and 3B and indirectly DNMT1. Blood, *113:* 6411-6418, 2009.

62. Li, Z., Hassan, M. Q., Jafferji, M., Aqeilan, R. I., Garzon, R., Croce, C. M., van Wijnen, A. J., Stein, J. L., Stein, G. S., and Lian, J. B. Biological functions of miR-29b contribute to positive regulation of osteoblast differentiation. J Biol Chem, *284:* 15676-15684, 2009.

63. Muniyappa, M. K., Dowling, P., Henry, M., Meleady, P., Doolan, P., Gammell, P., Clynes, M., and Barron, N. MiRNA-29a regulates the expression of numerous proteins and reduces the invasiveness and proliferation of human carcinoma cell lines. Eur J Cancer, *45:* 3104-3118, 2009.

64. Johnnidis, J. B., Harris, M. H., Wheeler, R. T., Stehling-Sun, S., Lam, M. H., Kirak, O., Brummelkamp, T. R., Fleming, M. D., and Camargo, F. D. Regulation of progenitor cell proliferation and granulocyte function by microRNA-223. Nature, *451:* 1125-1129, 2008.

65. Ramkissoon, S. H., Mainwaring, L. A., Ogasawara, Y., Keyvanfar, K., McCoy, J. P., Jr., Sloand, E. M., Kajigaya, S., and Young, N. S. Hematopoietic-specific microRNA expression in human cells. Leuk Res, *30:* 643-647, 2006.

66. Debernardi, S., Skoulakis, S., Molloy, G., Chaplin, T., Dixon-McIver, A., and Young, B. D. MicroRNA miR-181a correlates with morphological sub-class of acute myeloid leukaemia and the expression of its target genes in global genome-wide analysis. Leukemia, *21:* 912-916, 2007.

67. Mi, S., Lu, J., Sun, M., Li, Z., Zhang, H., Neilly, M. B., Wang, Y., Qian, Z., Jin, J., Zhang, Y., Bohlander, S. K., Le Beau, M. M., Larson, R. A., Golub, T. R., Rowley, J. D., and Chen, J. MicroRNA expression signatures accurately discriminate acute lymphoblastic leukemia from acute myeloid leukemia. Proc Natl Acad Sci U S A, *104:* 19971-19976, 2007.

68. Fazi, F., Rosa, A., Fatica, A., Gelmetti, V., De Marchis, M. L., Nervi, C., and Bozzoni, I. A minicircuitry comprised of microRNA-223 and transcription factors NFI-A and C/EBPalpha regulates human granulopoiesis. Cell, *123:* 819-831, 2005.

69. Fukao, T., Fukuda, Y., Kiga, K., Sharif, J., Hino, K., Enomoto, Y., Kawamura, A., Nakamura, K., Takeuchi, T., and Tanabe, M. An evolutionarily conserved mechanism for microRNA-223 expression revealed by microRNA gene profiling. Cell, *129:* 617-631, 2007.

70. Eyholzer, M., Schmid, S., Schardt, J. A., Haefliger, S., Mueller, B. U., and Pabst, T. Complexity of miR-223 regulation by CEBPA in human AML. Leuk Res, 2009.

71. Fazi, F., Racanicchi, S., Zardo, G., Starnes, L. M., Mancini, M., Travaglini, L., Diverio, D., Ammatuna, E., Cimino, G., Lo-Coco, F., Grignani, F., and Nervi, C. Epigenetic silencing of the myelopoiesis regulator microRNA-223 by the AML1/ETO oncoprotein. Cancer Cell, *12:* 457-466, 2007.

72. Pulikkan, J. A., Dengler, V., Paramangalam, P. S., Peer Zada, A. A., Muller-Tidow, C., Bohlander, S. K., Tenen, D. G., and Behre, G. Cell cycle regulator E2F1 and microRNA-223 comprise an autoregulatory negative feedback loop in acute myeloid leukemia. Blood, 2009.

73. Felli, N., Pedini, F., Romania, P., Biffoni, M., Morsilli, O., Castelli, G., Santoro, S., Chicarella, S., Sorrentino, A., Peschle, C., and Marziali, G. MicroRNA 223-dependent expression of LMO2 regulates normal erythropoiesis. Haematologica, *94:* 479-486, 2009.

74. Dixon-McIver, A., East, P., Mein, C. A., Cazier, J. B., Molloy, G., Chaplin, T., Andrew Lister, T., Young, B. D., and Debernardi, S. Distinctive patterns of microRNA expression associated with karyotype in acute myeloid leukaemia. PLoS One, *3:* e2141, 2008.

75. Haller, F., von Heydebreck, A., Zhang, J. D., Gunawan, B., Langer, C., Ramadori, G., Wiemann, S., and Sahin, O. Localization- and mutation-dependent microRNA (miRNA) expression signatures in gastrointestinal stromal tumours (GISTs), with a cluster of co-expressed miRNAs located at 14q32.31. J Pathol, *220:* 71-86.

76. Meng, F., Wehbe-Janek, H., Henson, R., Smith, H., and Patel, T. Epigenetic regulation of microRNA-370 by interleukin-6 in malignant human cholangiocytes. Oncogene, *27:* 378-386, 2008.

77. Maes, O. C., Sarojini, H., and Wang, E. Stepwise up-regulation of microRNA expression levels from replicating to reversible and irreversible growth arrest states in WI-38 human fibroblasts. J Cell Physiol, *221:* 109-119, 2009.

78. Tanaka, M., Oikawa, K., Takanashi, M., Kudo, M., Ohyashiki, J., Ohyashiki, K., and Kuroda, M. Down-regulation of miR-92 in human plasma is a novel marker for acute leukemia patients. PLoS One, *4:* e5532, 2009.

79. Lehmann, U., Hasemeier, B., Christgen, M., Muller, M., Romermann, D., Langer, F., and Kreipe, H. Epigenetic inactivation of microRNA gene hsa-mir-9-1 in human breast cancer. J Pathol, *214:* 17-24, 2008.
